# Unveiling the Multitarget Anti-Alzheimer Drug Discovery Landscape: A Bibliometric Analysis

**DOI:** 10.3390/ph15050545

**Published:** 2022-04-28

**Authors:** Anna Sampietro, F. Javier Pérez-Areales, Paula Martínez, Elsa M. Arce, Carles Galdeano, Diego Muñoz-Torrero

**Affiliations:** 1 Laboratory of Medicinal Chemistry (CSIC Associated Unit), Faculty of Pharmacy and Food Sciences, Institute of Biomedicine (IBUB), University of Barcelona, E-08028 Barcelona, Spain; annasampietropifarre@gmail.com (A.S.); paula14b@gmail.com (P.M.); e.martinezarce@ub.edu (E.M.A.); 2 Yusuf Hamied Department of Chemistry, University of Cambridge, Lensfield Road, Cambridge CB2 1EW, UK; fjp30@cam.ac.uk; 3 Department of Pharmacy and Pharmaceutical Technology and Physical Chemistry, Faculty of Pharmacy and Food Sciences, Institute of Biomedicine (IBUB), University of Barcelona, E-08028 Barcelona, Spain; cgaldeano@ub.edu

**Keywords:** multifactorial diseases, Alzheimer’s disease, polypharmacology, multitarget drugs, hybrids, target combinations, multitarget drug design, animal models

## Abstract

Multitarget anti-Alzheimer agents are the focus of very intensive research. Through a comprehensive bibliometric analysis of the publications in the period 1990–2020, we have identified trends and potential gaps that might guide future directions. We found that: (i) the number of publications boomed by 2011 and continued ascending in 2020; (ii) the linked-pharmacophore strategy was preferred over design approaches based on fusing or merging pharmacophores or privileged structures; (iii) a significant number of in vivo studies, mainly using the scopolamine-induced amnesia mouse model, have been performed, especially since 2017; (iv) China, Italy and Spain are the countries with the largest total number of publications on this topic, whereas Portugal, Spain and Italy are the countries in whose scientific communities this topic has generated greatest interest; (v) acetylcholinesterase, β-amyloid aggregation, oxidative stress, butyrylcholinesterase, and biometal chelation and the binary combinations thereof have been the most commonly pursued, while combinations based on other key targets, such as tau aggregation, glycogen synthase kinase-3β, NMDA receptors, and more than 70 other targets have been only marginally considered. These results might allow us to spot new design opportunities based on innovative target combinations to expand and diversify the repertoire of multitarget drug candidates and increase the likelihood of finding effective therapies for this devastating disease.

## 1. Introduction

Alzheimer’s disease (AD) is one of our greatest public health problems. Clinically, its effects on patients’ health are devastating. It is an irreversible, progressive neurodegenerative disorder with a very slow time-course, starting with a preclinical stage of 15–20 years, which is followed by a 3–6-year prodromal phase, before the appearance of dementia. At that point, it impairs thinking, reasoning, and memory, produces behavioral and personality changes, and eventually incapacitates the patients to perform daily life activities and inexorably leads to death. The prevalence of AD is huge. Indeed, it is the most prevalent neurodegenerative disease and the main cause of dementia, accounting for nearly 70% of all cases. Alzheimer’s Disease International estimated that in 2019, there were around 50 million people worldwide suffering from dementia, with this figure being expected to triple to reach 152 million by 2050 [1]. Considering its health effects and prevalence, not surprisingly, AD is one of the major causes of morbidity and mortality worldwide. According to estimates of the World Health Organization, in 2019 AD and other dementias ranked the seventh cause of death globally. Its impact is especially important in upper-middle-income and high-income countries, where AD ranked the eighth and the second leading cause of death, respectively, with the mortality figures being on the rise [2].

Another important consequence of the aforementioned facts and figures is the huge economic cost that is associated with AD, which currently reaches about USD 1 trillion per year, an amount that might double by 2030 [1]. Thus, AD imposes a huge societal and economic burden.

To a great extent, the terrible consequences of AD are due to the elusiveness of treatments that prevent, delay the onset, or slow down its progression. During the past two decades, only four drugs have been available for the treatment of AD: three acetylcholinesterase (AChE) inhibitors (donepezil, rivastigmine, and galantamine) and one antagonist of the glutamate NMDA receptors (memantine). These drugs only afford a modest temporary relief of the cognitive and neuropsychiatric symptoms of the disease [3]. In 2019, China’s National Medical Product Administration (NMPA) conditionally approved sodium oligomannate, an orally administered, marine algae-derived mixture of acidic linear oligosaccharides developed thanks to a public–private partnership (Ocean University of China and the company Green Valley (Shanghai, China)), which, at least in part, acts by reconditioning gut microbiota. This drug is currently undergoing Phase III clinical trials in USA and Europe, in the pursuit of a global regulatory approval [4,5,6]. Moreover, in June 2021, Biogen’s β-amyloid-directed monoclonal antibody aducanumab was approved in the USA after having generated much controversy about its actual clinical efficacy. Indeed, the manufacturer Biogen had reported a failure of aducanumab in pivotal Phase III clinical trials, but later it changed course based on a re-analysis of the clinical data and proceeded with the application for its approval, which was granted despite a recommendation against it of the Food and Drug Administration (FDA) advisory committee [7]. To add fuel to the fire, the European Medicines Agency (EMA) has recently recommended refusal of the marketing authorization for aducanumab, six months after its FDA approval, adducing lack of evidence of efficacy and safety concerns [8], thereby lowering the expectations around the eagerly awaited first disease-modifying anti-AD drug.

All in all, the high prevalence and the lack of efficacious treatments have led to a general concern about developing dementia, with 95% of the general population thinking they will inexorably develop it at some point in their lifetime, and to a quite well-spread notion that nothing can be done to prevent it, so that many people, including healthcare providers, wrongly think that dementia is part of normal aging [1]. In this scenario, the discovery of efficacious drugs that are able to prevent or alter the progress of neurodegeneration is a very urgent need. Notwithstanding that most efforts should be focused on the development of drugs able to modify the natural course of the disease, they should be accompanied by the search for alternative therapies that can more efficiently relieve the symptoms in patients in different stages of cognitive impairment, through mechanisms other than those underlying the pathogenesis of the disease. So far, the discovery and development of disease-modifying and better symptomatic drugs for AD remains very challenging and the continued clinical failures make it one of the therapeutic areas with the highest attrition rates [9].

Most of the 2173 clinical trials for AD performed before 2019 involved drug candidates designed to hit one target that seems to play a prominent role in AD pathogenesis, such as β-amyloid biology (22%), tau pathology (12%), mitochondrial dysfunction (17%), neurovascular mechanisms (8%), or neuroinflammation (5%), among others, while 19% were to address neurotransmitter deficits [10]. In January 2021, there were 126 candidates undergoing clinical trials (28 in Phase III, 74 in Phase II, and 24 in Phase I) for preclinical, prodromal, and AD dementia populations, thereby trying to address the equally important needs of prevention, delaying the onset, slowing down progression, and relieving the cognitive and neuropsychiatric symptoms. Out of the 126 clinical candidates, 104 (82.5%) were potential disease-modifying agents, 13 (10.3%) were symptomatic cognitive enhancers, and 9 (7.1%) were to reduce the neuropsychiatric symptoms. Candidates targeting β-amyloid (13.5%) or tau (8.7%) pathologies, inflammation/infection/immunity (15.1%), metabolism and bioenergetics (5.6%), or neurovascular mechanisms (4.8%) remained among the most pursued [11].

Unfortunately, essentially all single-target drug candidates that were designed to separately hit one of the aforementioned key underlying disease mechanisms, such as β-amyloid, tau, oxidative stress, neuroinflammation, metabolic and vascular risk factors, or other targets, have failed in clinical trials so far [12]. The etiology of AD is not completely understood, so maybe these candidates were not hitting the most convenient targets. Indeed, a large number of different signaling pathways have been identified, which contribute to the overall progression of AD [13], including β-amyloid, tau, genetic mutations, neurotransmitter signaling, mitochondrial dysfunction, endoplasmic reticulum stress, oxidative stress, neuroinflammation, the ubiquitin-dependent proteasome system, the autophagy and endosomal–lysosomal system, protein misfolding and molecular chaperones, insulin signaling, lipid/cholesterol metabolism, Ca^2+^ signaling, excitotoxicity, neurotrophic factors signaling, Wnt/β-catenin signaling, leptin signaling, blood–brain barrier and cerebrovascular dysfunction, and gut microbiota and nutrients, with each one involving a number of different potential therapeutic targets. This makes the selection of the signaling pathway and the specific target to be hit an extremely difficult task, especially considering that even though very valuable knowledge about the molecular mechanisms involved in these different signaling pathways has been gained, many questions on the key underlying mechanisms still remain unanswered [14].

A more likely explanation of the clinical failure of those anti-AD drug candidates lies in the fact that they were designed as single-target molecules, while the disease is multifactorial, with all the aforementioned altered signaling pathways playing an important role and being interconnected to form a complex pathological network where modulation of a single target results as simply insufficient. Indeed, drugs designed to selectively modulate a single biological target are unlikely to alter the progression of multifactorial diseases, whose complex pathological networks are provided with redundant and compensatory pathways that make them robust and resistant to a very partial modulation [15,16]. Conversely, the simultaneous modulation of several key signaling pathways through a multitarget therapy is increasingly regarded as more likely to inflict a greater spread and consistent damage to the pathological network, and, hence, to derive an efficacious treatment.

The most classical multitarget therapies rely on the use of combinations of several drugs, each one hitting a particular target, in different medications (drug cocktails) or in the same medication (fixed-dose combination). These types of drug combinations, which are being successfully used in complex diseases such as cancer and human immunodeficiency virus-1 (HIV-1), have also started to be considered in AD. Indeed, in 2014 the FDA approved a fixed-dose combination of the anti-AD drugs donepezil and memantine (Namzaric^®^), and other drug combinations are currently being tested in clinical trials, in most cases involving an AChE inhibitor and/or memantine [17]. Alternatively, a multitarget therapy can be accomplished through the use of single molecules that are able to interact with several biological targets, i.e., multitarget drugs. This approach involves just one drug in one medication, so it will benefit from greater simplicity in all steps of drug development (pharmacokinetics, pharmacodynamics, formulation, manufacturing, patent protection, regulatory issues, etc.) compared with multitarget therapies based on combinations of several drugs, will result in improved patient compliance arising from a simpler therapeutic regime relative to drug cocktails, and will avoid the risk of drug–drug interactions at different ADME phases (metabolism, elimination) that may arise when using drug combinations, resulting in lack of efficacy or toxicity issues. Indeed, the design and development of multitarget agents against multifactorial diseases in general [18], and against AD in particular [19,20,21], is a hot research topic, which is regularly the focus of dedicated theme issues and monographic sessions in medicinal chemistry journals and conferences.

Maybe the most critical aspect when trying to discover a multitarget drug is the selection of the biological targets to be hit, i.e., the combination of targets whose simultaneous modulation should afford additive or even synergistic effects to efficiently halt or slow down disease progression and/or efficiently alleviate the symptoms [17]. Not only is the selection of the target combination crucial to get the expected pharmacological outcome, but it also determines the design strategy.

**Figure 1:**
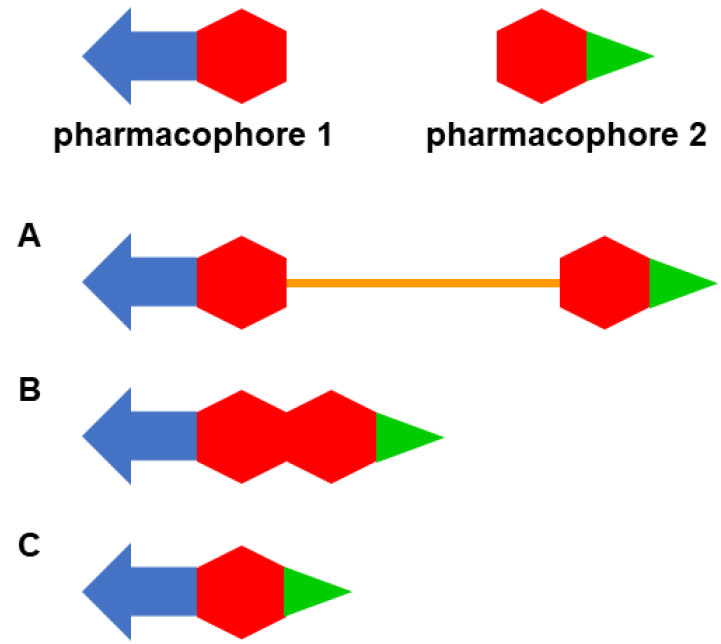
Design strategies used in the framework combination approach: linked hybrid ((**A**); the linker is colored in orange), fused hybrid (**B**); merged hybrid (**C**). The elements in different colors represent different structural fragments within each pharmacophore.

Multitarget drugs are usually designed through the framework combination approach, i.e., by combining two or more pharmacophoric moieties in the same molecule to enable the interaction of the resulting hybrid molecule with the selected biological targets [22,23]. The two pharmacophores of a multitarget molecule can be combined by linking or conjugating them through a tether, usually a chain (Figure 1A), by fusing them without any additional element (Figure 1B), or by merging them, overlapping some common structural motifs (Figure 1C).

The linked-pharmacophore strategy usually leads to rather large and lipophilic molecules, but it is usually very simple to apply and is especially useful to hit targets whose binding regions are buried deep inside the protein. In those cases, one pharmacophore of the hybrid may interact with the main binding site of the protein, whereas a linker with the appropriate geometry and length may place the second pharmacophore at a secondary binding site to enable a dual-site binding within that target, usually leading to increased potency [24]. When shifting from linked hybrids to fused and merged hybrids, additional (linker) or some common structural elements are avoided, which leads to reduced molecular weight and, in principle, more favorable physicochemical properties and drug likeness [25,26].

There exist some widely spread perceptions about the aforementioned aspects of multitarget anti-AD drug design:

(i) There is a clear feeling that the number of publications on multitarget anti-AD agents has steadily increased over the past one or two decades, especially since the publication in 2008 of a seminal review article by Cavalli, Bolognesi, Melchiorre, and coworkers [27], but the actual magnitude and evolution of the number of publications on this topic over the years has not been assessed so far.
(ii) A second, quite general notion is that most multitarget anti-AD compounds are designed to hit AChE and another target of interest [28]. Indeed, a large number of multitarget compounds feature pharmacophoric moieties of the approved AChE inhibitors [29,30,31,32], even though publications on multitarget anti-AD agents based on other pharmacophores, especially naturally occurring scaffolds such as β-carbolines [33], naphthoquinones and anthraquinones [34], or chalcones [35], just to name a few of those recently reviewed, are continuously appearing. Apart from AChE, other targets and pathogenic mechanisms that, intuitively, seem to have been commonly addressed when designing multitarget anti-AD compounds are glutamante NMDA receptors [36,37], β-amyloid and tau aggregation [38,39], glycogen synthase kinase 3β (GSK-3β) [40], biometal dyshomeostasis [41], monoamine oxidases (MAOs) [42], or oxidative stress [43].
(iii) It is usually assumed that the linked-pharmacophore strategy is the most popular design strategy, likely because AChE is thought to be the most commonly pursued target in multitarget anti-AD drug design and it is one of those proteins with binding sites buried at the bottom of a deep cavity.

All these perceptions are just intuitive, as none of these issues has been quantified so far. Thus, we do not have an accurate picture of the multitarget anti-AD drug design landscape. In particular, more precise knowledge of the biological targets and the target combinations that have been considered would be of paramount importance, as it would enable the identification of those targets and combinations that have been explored in more depth and, especially, those that have been barely investigated or not investigated at all, which would open new avenues in multitarget anti-AD drug design and increase the likelihood of finding efficacious treatments for this devastating disease.

Here, we provide a comprehensive analysis of the aforementioned and other aspects of multitarget anti-AD drug design landscape, through a bibliometric study of the publications on this topic over three decades, in the period 1990–2020. The primary aim of this study was (i) to quantify the frequency at which each biological target and each target combination were pursued in that period. As subsidiary objectives, we settled the analysis of (ii) the evolution of the number of publications throughout that period; (iii) the design strategy used; (iv) the type of biological study performed (in vitro/in vivo); and (v) the country where the works were performed.

## 2. Results and Discussion

### 2.1. Bibliographic Search

**Figure 2:**
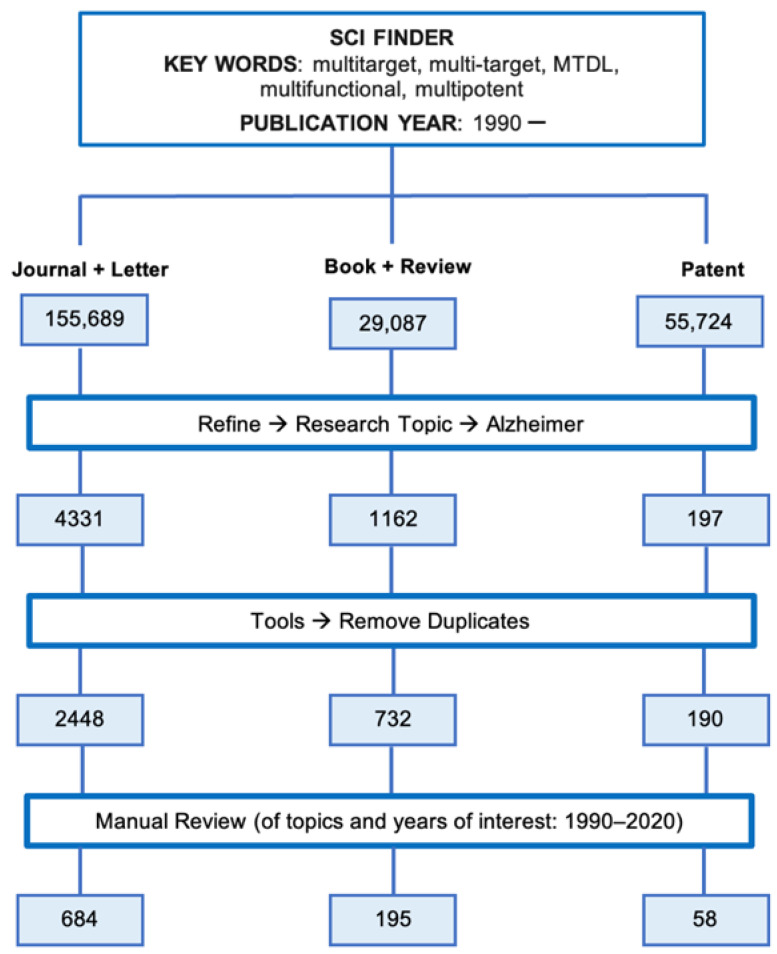
Bibliographic search workflow used for the bibliometric analysis.

To identify those publications dealing with multitarget anti-AD compounds, we performed a search in the SciFinder^®^ database using the workflow detailed in Figure 2.

The search began with the definition of the research topic. Different terms have been used in the literature to refer to the concept of multitarget agents, namely “multitarget”, “multi-target”, “multifunctional”, “multipotent”, and “MTDL” (multitarget-directed ligands). All these terms were considered as the “Research Topic”, using two additional “Advanced Search Fields”, namely “Publication Year(s)” and “Document type”. For the “Publication Year(s)” the period starting in 1990 (“1990-”) was settled and for the “Document Type”, either “Journal + Letter”, “Book + Review”, or “Patent” were considered in three parallel searches, which led to the identification of 155,689, 29,087, and 55,724 documents, respectively (Figure 2). In each one of these parallel search branches, the following procedure was applied: each search was first refined using the “Research Topic: Alzheimer” and then duplicated items were removed, to select only those documents dealing with anti-AD multitarget compounds. This led to a total amount of 2448 articles (“Journal + Letter”), 732 books and reviews, and 190 patents. These three sets of documents were distributed by the specific publication year (1990, 1991, …, 2020) and the resulting lists of documents were subjected to manual inspection, to select only those documents actually dealing with the design, synthesis, and pharmacological evaluation of multitarget anti-AD compounds. After following this workflow, a total of 684 research articles, 195 books and reviews, and 58 patents dealing with multitarget anti-AD agents published in the period 1990–2020 were identified.

With the information of the number of manually inspected and validated documents of each type that were published each year within the period 1990–2020, the actual magnitude of the number of publications on multitarget anti-AD compounds and their evolution over the years could be quantitatively determined. Moreover, focusing on the set of original research articles, the rest of the objectives of the planned bibliometric analysis were also addressed: the type and frequency of biological targets and target combinations pursued in the design of this type of compounds; the design strategies that have been employed; the type of biological assays to which these compounds have been subjected; and the countries where this approach is mostly pursued. To this end, those secondary research articles that deepened in the pharmacological study of a given multitarget compound or family whose design, synthesis, and multitarget profiling was previously reported in another research article were discarded, which resulted in 622 primary research articles. This was to ensure that a particular compound or class of compounds was considered only once in the bibliometric analysis. The results and discussions about all these points are included in the following subsections.

### 2.2. Evolution of the Number of Publications on Multitarget Anti-Alzheimer Compounds in the Period 1990–2020

**Figure 3:**
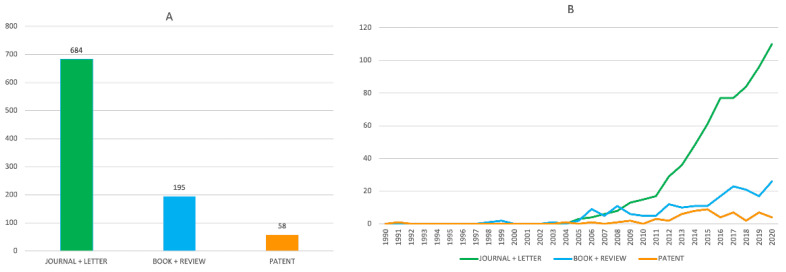
Total number (**A**) and evolution of the annual number (**B**) of original research articles (green bar and line), books and reviews (blue bar and line) and patents (orange bar and line) dealing with multitarget anti-AD compounds in the period 1990–2020.

According to the bibliographic search described before, a total of 937 publications on this topic appeared in the period 1990–2020, of which 684 were original research articles, 195 were reviews and books, and 58 were patents (Figure 3A). At first glance, these figures may not seem particularly striking if we consider that they appeared in a period of 30 years. However, the perception is completely different if we consider that most of these publications belong to a much narrower time-period. Indeed, until 2005 the publications on this topic were infrequent and it was around 2011 when they gained momentum, with an explosive growth, especially regarding research articles, which remained upwards in December 2020 (Figure 3B). Thus, out of the 684 research articles, 635 (93% of the total) were published in the last ten years (2011–2020) of the considered thirty-year period, which leads to a partial average of 63.5 papers per year, e.g., more than five papers per month during a period of 10 years, which is actually a striking figure. Regarding books and reviews and patents, 153 (78% of the total) and 52 (90% of the total), respectively, were published in the last ten years, i.e., on average more than 15 books and reviews and five patents per year, sustained over ten years.

### 2.3. Design Strategies of Multitarget Anti-Alzheimer Compounds in the Period 1990–2020

As previously mentioned, once the total number of publications on this topic were quantified, the rest of bibliometric analyses were performed focusing exclusively on those original research articles (622) that described each particular multitarget compound or class of compounds for the first time, so that the same compound or family was considered only once in the count. The type of strategy followed in the design of these compounds was analyzed, differentiating among linked, fused, and merged hybrids, and privileged structures (compounds that are not designed by combination of different pharmacophores but display inherent multitarget activity).

**Figure 4:**
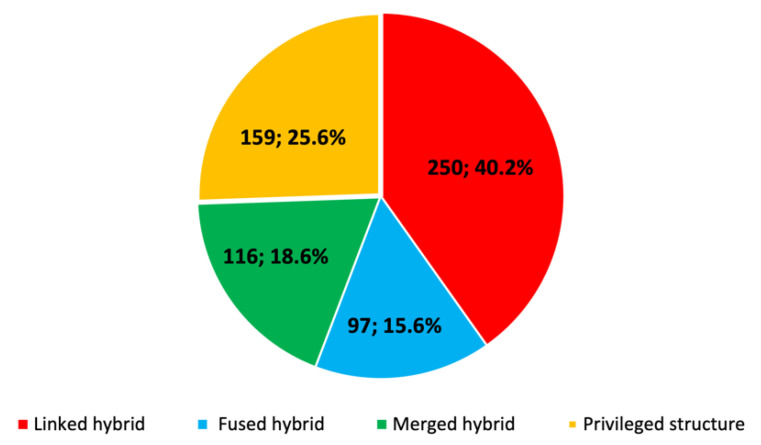
Strategies followed in the design of multitarget anti-AD compounds in the period 1990–2020.

As commonly believed, the major strategy that has been used so far in the field of multitarget anti-AD drug discovery involves the design of linked hybrids (40.2%) (Figure 4). Hybrids designed by fusing or merging different pharmacophores represent 15.6 and 18.6%, respectively, whereas privileged structures account for up to a quarter of the total (25.6%).

The design of linked hybrids is much easier than that of the other types of multitarget compounds. From a structural point of view, for the design of linked hybrids no special attention must be given to the pharmacophores to be combined, so that the attachment points of the linker to each pharmacophore and the functionality to be used for the linkages and within the linker are the main variables to be considered. Conversely, the design of fused and merged hybrids is much more challenging and depends largely on the structure of the pharmacophores to be combined, particularly on the presence of common motifs that can be eventually overlapped, which is not always possible.

From a biological point of view, linked hybrids may be especially useful to target proteins featuring a main binding site buried at the end of a deep cavity and a secondary binding site at the entrance of the cavity. In those cases, one pharmacophore is selected for each target to interact with the main binding site, whereas the second pharmacophore might interact with the secondary site, with the linker that joins the two scaffolds affording the appropriate geometry and distance. The resulting dual-site binding usually leads to increased potencies, as an added benefit apart from modulating two different targets. However, they are usually rather large and lipophilic molecules that are commonly regarded as less druglike than the smaller-sized fused and merged hybrids, inasmuch as they may have less favorable physicochemical and pharmacokinetic properties [25,26]. Indeed, some of them are non-compliant with Lipinski’s rules [44], so they might be expected to suffer from poor oral absorption. An additional alert with linked hybrids might come from their ability to cross the blood–brain barrier, a necessary attribute to exert their effects in the central nervous system (CNS). Indeed, if some linked hybrids do not comply with Lipinski’s rules, they might seem even farther to comply with the more stringent attributes that have been suggested to ensure brain permeation; for example, a molecular weight (MW) < 450 Da, calculated log P < 5, hydrogen bond donors (HBD) < 3, and hydrogen bond acceptors (HBA) < 7 [45] or even more strict attributes, with MW < 430 Da, clogP < 4, and HBD < 2 [46]. Notwithstanding these alerts, examples of linked hybrids have been described, which, despite not complying the aforementioned rules, can reach the CNS even at higher levels than the world’s leading anti-AD drug donepezil [47].

The ease of design and synthesis of linked hybrids and/or their usefulness to hit targets with extended gorges, including some targets widely pursued in multitarget anti-AD drug discovery, such as AChE, likely account for the popularity of this approach relative to the other particular design strategies. However, if the other design strategies, i.e., fused hybrids, merged hybrids, and privileged structures, are considered jointly, they represent the majority (60%). In these cases, there should not be especial issues with their physicochemical and pharmacokinetic properties as they tend to be smaller, druglike molecules.

### 2.4. Pharmacological Profiling of Multitarget Anti-Alzheimer Compounds in the Period 1990–2020

**Figure 5:**
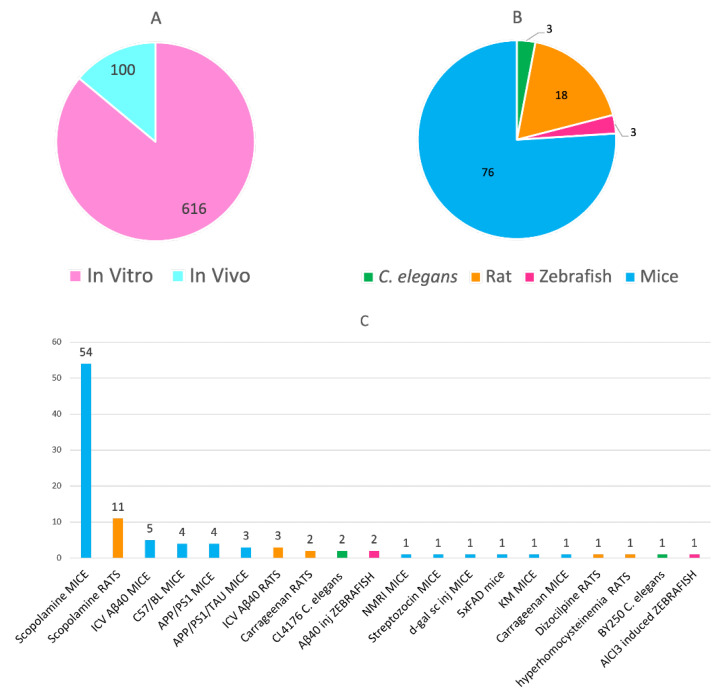
Number of in vitro and in vivo studies (**A**); type of animals (**B**) and specific models (**C**) used for the pharmacological evaluation of multitarget anti-AD compounds in the period 1990–2020.

The vast majority of the analyzed works (616 out of 622) include an in vitro evaluation of the multitarget compounds towards their multiple biological targets (Figure 5A), usually by enzyme inhibition and radioligand binding assays. Indeed, this is a quite expectable result, considering that this analysis was performed from the original research articles that described each class of multitarget anti-AD compounds for the first time. Globally, a total of 100 in vivo efficacy studies have been reported in the studied period (Figure 5A), which followed the in vitro characterization of the compounds. The number of reported in vivo efficacy studies with this type of compounds was relatively scarce just a few years ago [48], but it has almost tripled from 2017 (28 in vivo studies in the period 1990–2016 vs. 72 from 2017 to 2020), indicating an increased interest to advancing multitarget anti-AD agents to preclinical and clinical testing.

Out of the 100 in vivo studies, 76 (76% of the total) were performed using different mouse models (Figure 5B). Rats, *Caenorhabditis elegans*, and zebra fish have been also used as model animals, albeit to a minor extent (Figure 5B). Regarding the specific animal model employed for the in vivo efficacy studies, the most widely used, by far, is the model of amnesia induced by scopolamine in wild-type mice and rats (Figure 5C). Even if this model may be useful to assess the effects of the compounds on learning and memory, it does not recapitulate other important underlying mechanisms of AD. To this end, transgenic animals, such as APP/PS1, APP/PS1/tau, and 5 × FAD mice, may be more useful, even though they have been clearly less used so far, likely because they are much more expensive and not so easily available.

Many of these in vivo studies included groups of animals that were treated with one or several single-target individual reference drugs, for comparison with the group of animals treated with the multitarget compound. As reference drugs, in most cases the marketed anti-AD drugs (donepezil, rivastigmine, galantamine, memantine) and tacrine were used, but the AChE inhibitor huperzine A and other drugs or clinical candidates with other specific mechanisms of action related to the studied multitarget compound, such as the antioxidant trolox, the metal chelating agent clioquinol, the MAO-B inhibitor pargyline, the nootropic agent piracetam, or the PDE4 inhibitors rolipram and roflumilast have also been used. Interestingly, in many of these studies the multitarget compound performed better than the reference drug in terms of improved cognition and levels of certain biomarkers of relevance for the putative mechanisms of the multitarget agent. This provides experimental evidence that the multitarget compound is more efficacious that the reference single-target drug in a head-to-head comparison. From these results it could be inferred that the superior in vivo effects of the multitarget compounds arise from the expected simultaneous modulation of several biological targets, but a robust demonstration of the latter point would require more sophisticated studies, which are usually not affordable for many academic groups, in which most of this research is carried out.

### 2.5. Geographical Origin of the Works on Multitarget Anti-Alzheimer Compounds in the Period 1990–2020

**Figure 6:**
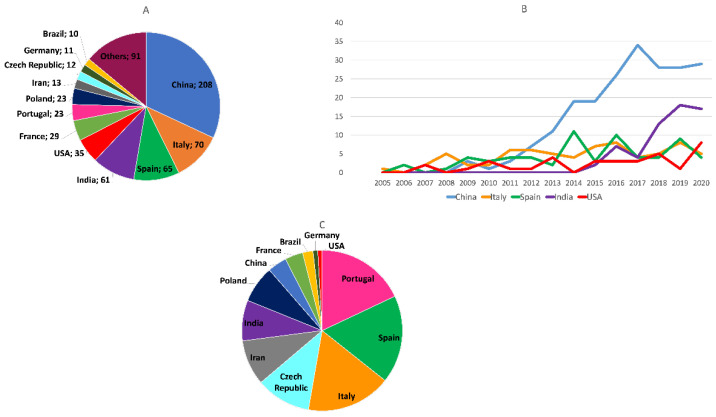
(**A**) Geographical origin of the articles on multitarget anti-AD compounds in the period 1990–2020, considering the total number of published articles; (**B**) evolution of the total number of original research articles on multitarget anti-AD compounds coming from the top five contributor countries in the period 1990–2020; (**C**) geographical origin of the articles on multitarget anti-AD compounds in the period 1990–2020, considering the total number of articles normalized by the number of researchers in each country.

The geographical origin of the research articles on multitarget anti-AD compounds published from 1990 to 2020 was analyzed by considering the country of the institution to which the corresponding author(s) of each article was affiliated. According to the total number of published articles, so far, China has been the major contributor to the publications on this topic, followed by Italy, Spain, India, USA, France, Portugal, Poland, Iran, the Czech Republic, Germany, and Brazil, considering the countries with 10 or more contributions (Figure 6A). Publications from China started to appear later than those from other countries such as Italy, Spain, and the USA, but only a few years thereafter, they experienced a very significant growth, approximately from 2012 onwards (Figure 6B), coinciding with and contributing to the global marked increase in the number of publications on multitarget anti-AD compounds in the last decade. Publications coming from India have been much more concentrated in time, as they began as late within the studied period as in 2014, but since then they increased significantly, so that India became the second major contributor at the end of the considered period (Figure 6B).

As an indicator of the relative interest of the scientific community of each country toward multitarget anti-AD compounds, the total number of publications on this topic by each contributing country was normalized by its number of researchers, which was calculated from the number of researchers in R&D per million people [49] and the total population of each country. According to the normalized number of publications, Portugal, Spain, and Italy are the countries in whose scientific communities multitarget anti-AD agents have been the focus of most intensive research, followed by the Czech Republic, Iran, India, Poland, China, France, Brazil, Germany, and the USA, considering the countries with 10 or more contributions (Figure 6C).

### 2.6. Biological Targets and Target Combinations Hit by Multitarget Anti-Alzheimer Compounds in the Period 1990–2020

The selection of the multiple biological targets to be hit is likely the most critical aspect in multitarget drug discovery, as it determines the occurrence of additive or even synergistic effects that are to alter the course of the disease and/or to lead to a more effective relief of the symptoms. Thus, the identification and quantification of the frequency at which the different biological targets and pathological events and their combinations have been pursued in the design of multitarget anti-AD agents constitute the main aim of this bibliometric analysis, especially because identification of those targets and combinations that have not been addressed so far or that have been very scarcely explored might bring new opportunities to advance the field.

**Figure 7:**
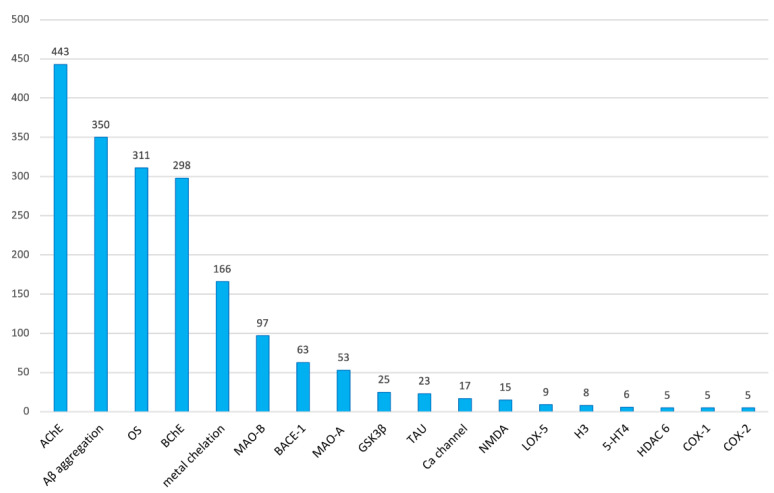
Biological targets that have been pursued in 5 or more research articles on multitarget anti-AD compounds in the period 1990–2020 and number of articles in which they have been considered.

To this end, for each research article, the individual biological targets that were hit and their respective binary combinations were annotated. As commonly believed, AChE is clearly the most pursued biological target in multitarget anti-AD compounds. AChE was targeted in 443 (71.2%) out of the total of 622 research articles considered (Figure 7). Together with AChE, the aggregation of the β-amyloid peptide (Aβ, 56.3%), oxidative stress (OS, 50.0%), butyrylcholinesterase (BChE, 47.9%), and metal chelation (26.7%) are within the top five preferred targets in multitarget anti-AD drug design. Indeed, having been considered in one to three quarters of all the published research articles, all of them can be regarded as widely explored targets in multitarget anti-AD drug discovery.

Monoamine oxidase B (MAO-B, 15.6%) and BACE-1 (10.1%) are also relatively common targets of multitarget anti-AD compounds. Other targets that appear at least in five articles but with less than 10% of representativity are monoamine oxidase A (MAO-A, 8.5%), GSK-3β (4.0%), tau aggregation (3.7%), calcium channels (2.7%), NMDA receptors (2.4%), lipooxigenase-5 (LOX-5, 1.4%), histamine receptors H3 (1.3%), serotonin receptors 5-HT4 (1.0%), histone deacetylase 6 (HDAC 6, 0.8%), cyclooxygenase 1 (COX-1, 0.8%), and cyclooxygenase 2 (COX-2, 0.8%) (Figure 7).

**Figure 8:**
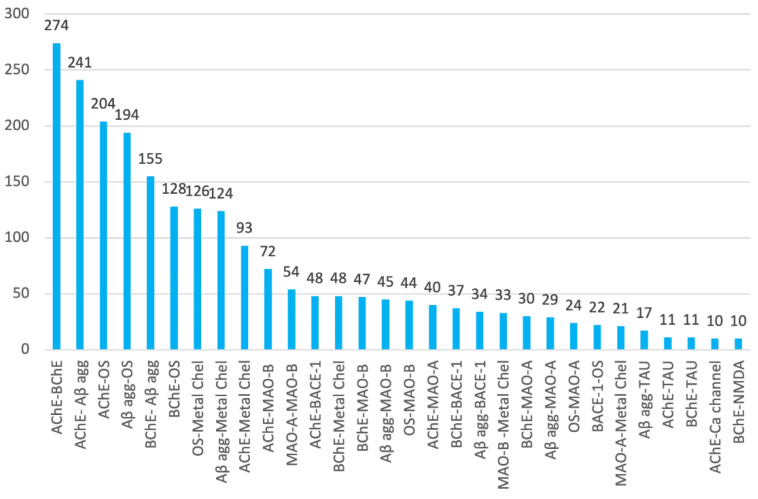
Combinations of biological targets that have been pursued in 10 or more research articles on multitarget anti-AD compounds in the period 1990–2020 and number of articles in which they have been considered.

If AChE, Aβ aggregation, oxidative stress, BChE, and metal chelation are the most commonly pursued individual targets of multitarget anti-AD compounds, their respective binary combinations are also the most widely considered. Thus, the top ten target combinations are AChE–BChE (in 44.1% of research articles), AChE–Aβ aggregation (38.7%), AChE–oxidative stress (32.8%), Aβ aggregation–oxidative stress (31.2%), BChE–Aβ aggregation (24.9%), BChE–oxidative stress (20.6%), oxidative stress–metal chelation (20.3%), Aβ aggregation–metal chelation (19.9%), AChE–metal chelation (15.0%), and AChE–MAO-B (11.6%) (Figure 8). AChE and BChE exert the same (patho)physiological effect, i.e., they catalyze the hydrolysis of the neurotransmitter acetylcholine in the CNS, which in the case of AD patients aggravates the brain cholinergic deficit that leads to the cognitive symptoms of the disease. Thus, targeting of AChE and BChE shares the same rational basis, which likely accounts for the fact that their combination is the one most present in multitarget anti-AD compounds. Similarly, Aβ aggregation, oxidative stress, and biometal dyshomeostasis seem to be intertwined processes, which could also explain the high frequency of their combinations.

Paralleling the frequency of the individual targets, combinations of MAO-B, MAO-A, and BACE-1 with the top five major targets are also relatively common, while combinations involving tau aggregation, calcium channels, and NMDA receptors have been less explored (Figure 8), and binary combinations involving GSK-3β are even scarcer.

**Figure 9:**
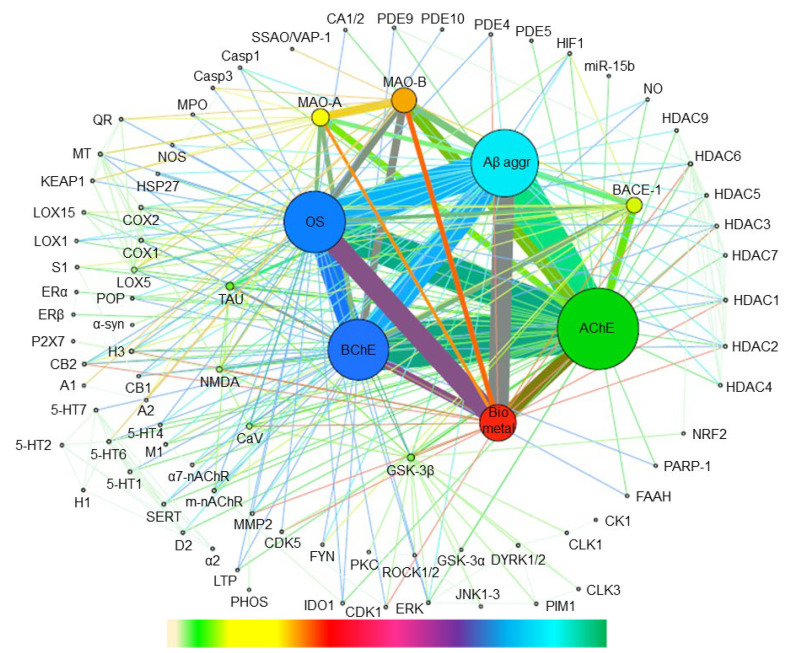
Mapping of the binary combinations of biological targets that have been pursued in multitarget anti-AD compounds in the period 1990–2020. For the abbreviations of the names of the biological targets, see Table 1. Individual targets and binary combinations between targets appear as nodes and edges connecting each pair of nodes, respectively. The size of the nodes and the thickness of the edges are proportional to the number of times that each individual target appears in a binary combination and to the number of times that each binary target combination has been pursued, respectively. Color codes have been used to distinguish ranges of frequency in targets and target combinations: gradient bar chart (down), from lower frequency (left) to higher frequency (right).

To more visually highlight the binary combinations of all the targets that have been considered so far in multitarget anti-AD drug discovery, the annotated target combinations were mapped using the open-source software Gephi [50], which is especially suited to revealing associations within networks and patterns of biological data. A graph was built, where individual targets are represented as nodes while binary combinations between targets appear as the edges connecting each pair of nodes (targets), with the size of the nodes and the thickness of the edges being proportional to the frequency at which each target appears in a binary combination and each binary target combination has been pursued, respectively (Figure 9).

The previously discussed trends about the most commonly pursued individual targets and binary target combinations are very evident upon inspection of Figure 9. Combinations among AChE, Aβ aggregation, oxidative stress, BChE, and biometal chelation have been vastly explored. To a lower extent, combinations based on MAO-B, MAO-A, and BACE-1 have also received considerable attention. However, it can be highlighted that targets with a putative important role in AD pathogenesis such as tau aggregation and GSK-3β, involved in tau pathology, or NMDA receptors and voltage-gated calcium channels, involved in calcium loading-induced neurotoxicity, have been much less explored by a significant amount. Likewise, combinations based on other more than 70 different targets, depicted in the periphery of the graph, have barely been explored, thereby leaving room for a large number of new target combinations that remain to be interrogated.

Indeed, combinations GSK-3β/BACE-1, MAO-B/biometals, phosphodiesterases (PDEs)/biometals, and BACE-1/cyclooxygenases (COXs) have been recently suggested as promising for multitarget anti-AD agents [28,51,52,53].

Histone deacetylases (HDACs) seem to play a prominent role in neurodegenerative diseases, and their modulation may rescue synaptic plasticity, memory, and learning through interconnections with different signaling pathways, such as tau biology, oxidative stress, neuroinflammation, and autophagy [54]. Even if some HDAC-hitting multitarget compounds have been described, the occurrence of HDAC-based combinations is almost anecdotic, so they should be explored in much more detail, as recently suggested, for example, for the combinations HDACs/GSK-3β and HDACs/PDEs [53].

A group of biological targets whose combinations are clearly underrepresented in the multitarget anti-AD drug discovery landscape are kinases. Deregulation of kinases, such as GSK-3β, FYN kinase, and dual-specificity tyrosine phosphorylation-regulated kinase 1A (DYRK1A), seems to be directly linked to several neurodegenerative diseases. Thus, these kinases, which are interconnected with different signaling pathways, are considered to play a key role in the neurokinome so that they may constitute emerging targets for future multitarget compounds [55]. Rho kinases (ROCKs) are the main effectors of RhoA, a member of the Rho family of GTPases that has important roles in synaptic processes. Thus, the RhoA/ROCK pathway has a central role in the pathogenesis of a number of CNS diseases, including AD. Indeed, ROCKs regulate proteins involved in cytoskeleton processes, destabilize microtubules, and prevent neurite elongation upon direct tau phosphorylation, thereby triggering synaptic dysfunction in AD. Thus, ROCKs seem to be an emerging target in AD, whose combinations should be explored to a greater extent in the field of multitarget anti-AD drugs [56].

Modulation of receptors involved in different neurotransmitter systems also represents an emerging mechanism for multitarget anti-AD agents. Particularly, combinations involving the modulation of serotonin 5-HT4 and 5-HT6, histamine H3, dopamine D3, and GABA receptors might be particularly useful to achieve enhanced efficacy against the cognitive and neuropsychiatric symptoms [12,28,53,57,58]. Cannabinoid receptors 1 and 2 (CB1, CB2) are components of the endocannabinoid system with a key role in AD, as they are involved in the regulation of cognition, memory, and neuroinflammatory processes [59]. Particularly, activation of CB2 receptors leads to suppression of microglia activation and proinflammatory cytokines and amelioration of synaptic plasticity, memory, and learning [60]. Thus, CB2 agonism should be the focus of more research efforts when designing multitarget anti-AD compounds [53]. Like the aforementioned different types of receptors, sigma-1 receptors (S1), which are also abundant in the CNS, have received very little attention so far in multitarget anti-AD drug design. Modulation of S1 receptors affords neuroprotective effects and may potentiate the effects of other receptors, rescuing endoplasmic reticulum stress and mitochondrial dysfunction. Thus, the combined modulation of S1 receptors together with dopamine, muscarinic, and glutamate NMDA receptors have been proposed as a new avenue in multitarget anti-AD drug discovery [61].

Many other innovative target combinations can be envisaged upon inspection of Figure 9. The repertoire of new combinations can be further expanded by considering biological targets that have not been considered so far in the design of multitarget compounds, but which have been recently reported to exert a key role in AD pathogenesis.

Autophagy might be such target, as it is involved in the clearance of Aβ and seems to be interconnected with many other signaling pathways. Indeed, impaired autophagic regulation leads to impaired Aβ clearance and increased neuronal death, so that upregulation of autophagy should be the focus of more intensive research [14].

Taking advantage of the existence of certain types of neurotransmitter receptors (5-HT3 and 5-HT4 receptors, and α7, α9, and α10 nicotinic receptors) and calcium channels in the mitochondrial membrane, new multitarget anti-AD agents could be designed to hit these targets in combination with mitochondrial targets, such as enhancement of mitochondrial bioenergetic potential, activation of mitochondrial biogenesis, modulating peroxisome proliferator-activated receptors (PPARs), transcription coactivators such as PPARγ coactivator-1 (PGC-1), nuclear transcription factors such as nuclear respiratory factors 1 and 2 (NRF-1 and NRF-2), and the mitochondrial transcription factor A (TFAM), or modulation of mitochondrial permeability transition (MPT) pores [61,62].

Epigenetic targets other than HDACs, such as lysine-specific demethylase 1A (KDM1A or LSD1), might be also considered in the design of new multitarget agents, as its inhibition may rescue memory deficits. Transglutaminase 2 (TG2), an enzyme involved in the cross-linking of critical proteins of AD pathology, whose inhibition leads to decreased oxidative stress-induced neuronal cell death, seems to be another target of interest for new multitarget compounds. Likewise, combinations including microglial targets, which seem to be critically involved in the early stages of AD, such as purinergic P2X and P2Y receptors, adenosine P1 receptors, toll-like receptors TLR2 and TRL4, microglia and fractalkine (CX3CL1)/fractalkine receptor (CX3CR1), receptor-interacting serine/threonine-protein kinase 1 (RIPK1), and triggering receptor expressed in myeloid cells 2 (TREM2) should be more intensively explored [51,63].

Epoxyeicosatrienoic acids (EETs) are metabolites of arachidonic acid that, among other effects, reduce inflammation and oxidative stress. In turn, EETs are metabolized by the enzyme soluble epoxide hydrolase (sEH) by opening of their epoxide ring to the corresponding diols, which abolishes their beneficial effects. Because inhibition of sEH results in anti-inflammatory effects, this enzyme has been considered in the design of different classes of multitarget agents to treat inflammation and pain [64,65,66]. Very interestingly, sEH is upregulated in the brains of AD patients and has been recently validated as a new biological target of interest for the treatment of AD [67,68,69,70]. Indeed, sEH inhibitors have been shown to be able to rescue cognitive deficits and to reduce neuroinflammation and Aβ and tau pathologies in different mouse models of AD [67]. Thus, sEH has emerged as a very promising target to be pursued in multitarget anti-AD drug discovery.

Ideally, mapping the interactome network of AD would be the most valuable tool to shed light on disease mechanisms and disease vulnerabilities, and to reveal key targets to be hit, thereby guiding multitarget drug design. Important endeavors are being carried out to decipher the AD interactome, but so far this remains very challenging, so that our knowledge is still very limited [71,72,73,74,75]. Even so, some important findings are being generated from this type of study. For example, in a recent work to map the interactome of neurodegenerative diseases, the abnormal aggregation of two proteins in the brain of AD patients has been identified as a key contributor to AD pathogenesis; namely, the aggregation of ataxin-1 (ATXN1), a DNA-binding protein that seems to be involved in gene expression regulation, and that of megacaryoblastic leukemia protein-1 (MKL1), a transcriptional coactivator for the serum response factor that plays a critical role in dendritic spine maturation. A loss of function of these proteins after abnormal aggregation in the brain might contribute to AD pathogenesis and progression [72]. Thus, aggregation of ATXN1 and MKL1 should be considered when designing new target combinations in multitarget anti-AD drug discovery.

Overall, a large number of opportunities for new target combinations in multitarget anti-AD drug discovery become evident upon inspection of the current landscape (Figure 9), which will be expanded by the emergence of new targets with an important role in AD pathogenesis. This makes a very judicious selection of the targets to be hit even more necessary. The targets should be likely to afford additive or synergistic effects and should be involved in contemporaneous pathologies within the neurodegenerative process [12]. In this light, Bolognesi and coworkers have proposed the following flowchart to validate a given target combination for a de novo multitarget approach: (i) identification of selective and nearly equipotent reference compounds for each selected target; (ii) demonstration of lack of cytotoxicity of the combination of reference compounds; (iii) demonstration of additive or synergistic effects in cell models that recapitulate AD pathological features; and (iv) demonstration of beneficial effects in an in vivo proof-of-concept using a suitable animal model [17].

Like any other field of drug discovery and development, the progression of investigational multitarget anti-AD compounds to the clinic must necessarily involve the participation of pharmaceutical companies. Unfortunately, this does not seem to be the case so far. Maybe the fact that many multitarget anti-AD compounds are rather large lipophilic molecules, i.e., out of Lipinski’s rules, or that many of them target AChE, a widely explored target whose inhibitors are labeled as symptomatic, non-disease-modifying agents results as a deterrent for an industry with a high inertia to pursue highly selective single-targeted drugs in one of the fields perceived as riskier. As discussed above, we and many others have demonstrated that some of these compounds can really enter the brain and exert central effects, despite not being compliant with Lipinski’s rules. We and many others really think AChE is still a target of interest for AD treatment. In the absence of biomarkers of very early disease stages, it is quite likely that AD treatment will have to address a certain degree of cognitive impairment, apart from trying to halt disease progression as much as possible. In this scenario, AChE-based multitarget agents may be of interest.

Anyway, we think it is time to be pragmatic and try to engage pharmaceuticals companies for development or co-development of multitarget agents that may result attractive for them. To ensure novelty and inventiveness and, hence, patentability of the projects on multitarget agents, we think we should focus on innovative combinations of targets that are contemporaneously involved in early stages of AD so that, at the same time, we avoid the use of some vastly used chemical scaffolds as those purposed for modulation of AChE, for example. Especially interesting might be those combinations of targets that are involved in the pathogenesis of several diseases, apart from AD, as this might increase the market opportunities of the multitarget drug, and hence, its attractiveness for pharmaceutical companies. In this light, we consider especially interesting targeting neuroinflammation and/or tau pathology with multitarget drugs. These events, together with Aβ pathology, are confluent in a crucial pathogenic event in AD, which is synaptic dysfunction and loss that eventually lead to cognitive impairment [76].

To this end, in our opinion, sEH might be a central biological target for the design of different classes of innovative multitarget agents against a variety of neurologic disorders that have neuroinflammation as a key pathogenic mechanism, including AD. As previously mentioned, sEH inhibitors reduce neuroinflammation and Aβ and tau pathologies and display cognition-enhancing effects in different mouse models of AD [67]. Very recently, we have reported the first class of dual inhibitors of sEH/AChE [77]. The design rationale was that inhibition of AChE, apart from leading to cognitive improvement, increases the levels of the neurotransmitter acetylcholine, which, in turn, may promote the metabolism of arachidonic acid to the anti-inflammatory EETs, i.e., the same effects that arise from sEH inhibition, by a different mechanism. This might result in synergistic or additive effects against neuroinflammation. Indeed, low-dose oral administration of the lead compound to SAMP8 mice rescued memory, synaptic plasticity and neuroinflammation in these AD model mice. Beyond AChE, we think it would be of great interest to combine sEH with other targets with a role in inflammation mediated by arachidonic acid metabolites, such as COX2 and LOX5, and with PPARγ receptors, which are expressed in neurons, where they are involved in inflammation, oxidative stress, and neuronal death [78]. On the one hand, a few examples of dual inhibitors of sEH/COX2 [79], sEH/LOX5 [80], and dual sEH inhibitors/PPARγ activators [81] have been developed for non-neurological applications, in which the multitarget compounds displayed positive effects against inflammation and oxidative stress. On the other hand, COX2 and LOX5 have been already used, albeit only very marginally, and PPARγ receptor has been suggested to be used in the design of multitarget anti-AD agents, but not in combination with sEH. Thus, we think that sEH/COX2, sEH/LOX5, and sEH/PPARγ are innovative combinations which hold great potential for the efficacious treatment of neurodegenerative disorders that occur with a key component of neuroinflammation, such as AD.

Besides AD, tau protein plays a key pathogenic role in a number of neurodegenerative disorders, called tauopathies, which also include Pick disease, progressive supranuclear palsy, and chronic traumatic encephalopathy (CTE), among others. These disorders are characterized by abnormal deposition of tau protein in the brain [82,83]. As previously mentioned, despite the relevance of tau pathology, it has been clearly less explored than other seemingly less relevant targets when designing multitarget anti-AD compounds. The enzyme p38 mitogen-activated protein kinase α (p38α) is expressed in neurons, where it is involved in tau localization and neuronal plasticity. Indeed, p38α seems to be playing a role in the aforementioned confluence of tau pathology, Aβ pathology, neuroinflammation, and synaptic dysfunction. In microglia and astrocytes, p38α regulates brain inflammation and it is a critical contributor to the toxicity of Aβ, tau, and inflammation to synapses, whereas p38α deficiency or inhibition is beneficial in AD animal models [84,85]. Thus, we suggest that p38α is considered a promising central node to be hit in combination with other kinases involved in tau pathology, such as GSK-3β, CDK5, CK1/2, DYRK1A and FYN, with prominent roles in tau phosphorylation [55,86].

As a final remark, we suggest that, whenever possible, the in vivo efficacy studies with the new multitarget anti-AD lead compounds include a group of treatment with a combination of the two reference drugs that are representative of the pursued mechanisms of action, besides the corresponding groups treated separately with each individual reference drug. Certainly, this can be cost-prohibitive is some cases, but demonstration of superior efficacy of the multitarget drug over the individual reference drugs and the combination thereof should hopefully be sufficiently convincing and appealing to attract the attention of pharmaceutical companies, facilitating the eagerly awaited massive entry of this class of compounds in clinical trials for AD and other neurodegenerative diseases.

## 3. Materials and Methods

### 3.1. Bibliographic Search

The bibliographic search was performed using the SciFinder® database, using as the “Research Topic” the terms “multitarget”, “multi-target”, “multifunctional”, “multipotent”, and “MTDL”. The “Publication Year(s)” and “Document type” were used as two additional “Advanced Search Fields”, settling “1990-” for the “Publication Year(s)” and “Journal + Letter”, “Book + Review”, or “Patent” for the “Document Type”. Each search was refined using the “Research Topic: Alzheimer”, duplicated items were removed, and the resulting sets of documents were distributed by the specific publication year and manually inspected to select the documents actually dealing with multitarget anti-AD compounds.

### 3.2. Creation of the Gephi Graph

To collect the information that was necessary for the creation of the Gephi graph, we prepared a list of biological targets that were hit by the multitarget anti-AD compounds described in the period 1990–2020 and a second list collecting the different binary target combinations and the number of times that each particular target combination had been pursued. With this information, two tables were built. In one table, consecutive numbers were assigned to the different biological targets (Table S1 of the Supplementary Materials). In the second table, the different binary combinations were introduced, identifying each target with the assigned number and considering each combination as unidirectional, e.g., it is not important whether the combination goes from 1 to 2 or from 2 to 1 (Table S2 of the Supplementary Materials).

The two tables were converted into .CSV files, which were uploaded to the Gephi 8.2 program, identifying these tables as the nodes table (targets) or the edges table (binary target combinations). After uploading these files, a graph was generated, showing all nodes and edges in the same size. To further process the graph, in the network overview section, the calculation average degree and average degree with weights was executed (Figure S1 of the Supplementary Materials) so that nodes and edges appeared in different sizes, which were proportional to the number of times that each target (node) and binary combination (edge) had been pursued in all the annotated target combinations. A color scale was used to more visually distinguish the different size ranges in nodes and edges.

## 4. Conclusions

Bibliometric analysis of the publications on multitarget anti-AD agents in past three decades (1990–2020) has revealed the following trends:

- It was not until one decade ago (around 2011) that the number of publications on this topic experienced an explosive growth. This upward growth remained at the end of the studied period.
- It is very likely that, because of their ease of design and synthesis and the appropriateness to hit targets with binding sites that extend along deep gorges, commonly pursued in multitarget anti-AD compounds such as AChE, linked hybrids are more common than the smaller-sized fused or merged hybrids and privileged structures, even though the latter should be preferable in terms of better physicochemical and pharmacokinetic properties.
- In addition to the mandatory in vitro evaluation towards the multiple targets, a significant number of in vivo studies have been performed to assess the efficacy of multitarget anti-AD compounds, especially from 2017 (72 in vivo studies out of a total of 100 in the whole period), indicating a clear willingness to advance these candidates to preclinical and clinical testing. By far, the model of amnesia induced by scopolamine in wild-type mice and, to a minor extent, in rats, has been the most widely used animal model, which enables the evaluation of behavioral effects but not the impact on the underlying disease mechanisms. When affordable, other models which better recapitulate the different pathologies of AD, e.g., suitable transgenic animals, should be used to more consistently assess the in vivo efficacy of the multitarget compounds.
- Within the overall studied period, China has been the major contributor to the publications on this topic. Italy, Spain, India, the USA, France, Portugal, Poland, Iran, and the Czech Republic complete the list of the top ten most contributing countries. At the end of the period, China and India were clearly the countries with a greatest productivity. When the number of publications was normalized by the number of researchers of each country, we found that Portugal, Spain, and Italy are the countries in whose scientific communities multitarget anti-AD agents have attracted more interest, followed by the Czech Republic, Iran, India, Poland, China, France, Brazil, Germany, and the USA.
- AChE is clearly the most commonly pursued biological target in multitarget anti-AD compounds, appearing in up to 71.2% of all the research articles on this topic. In the list of the top ten most common targets, we also found Aβ aggregation (56.3%), oxidative stress (50.0%), BChE (47.9%), metal chelation (26.7%), MAO-B (15.6%), BACE-1 (10.1%), MAO-A (8.5%), GSK-3β (4.0%), and tau aggregation (3.7%). Eighty other different targets, including different subtypes and isoforms of some receptors and enzymes, were also pursued in the multitarget compounds developed in the studied period, but most of them only very sporadically.
- Paralleling the frequency at which individual biological targets have been considered in multitarget anti-AD drug design, the binary combinations of the most common targets have been the most widely pursued. In the list of the top ten binary target combinations, we found AChE–BChE (in 44.1% of research articles), AChE–Aβ aggregation (38.7%), AChE–oxidative stress (32.8%), Aβ aggregation–oxidative stress (31.2%), BChE–Aβ aggregation (24.9%), BChE–oxidative stress (20.6%), oxidative stress–metal chelation (20.3%), Aβ aggregation–metal chelation (19.9%), AChE–metal chelation (15.0%), and AChE–MAO-B (11.6%).

With all this information, it becomes apparent that combinations based on targets with a key role in AD pathogenesis such as tau aggregation, GSK-3β, NMDA receptors, and voltage-gated calcium channels have been significantly much less explored, and combinations involving more than other 70 different targets have barely been explored.

Notwithstanding the interest of the most commonly pursued targets and combinations, innovative combinations based on many other less-explored targets, such as those appearing in the periphery of the graph, and new emerging targets should be studied in more depth. In this light, without underestimating many other potential target combinations, we suggest that the combinations of sEH with other targets that mediate neuroinflammation, such as COX2, LOX5 and PPARγ, and the combinations of p38α with kinases involved in tau phosphorylation, such as GSK-3β, CDK5, CK1/2, DYRK1A, and FYN, are especially worthy to be explored for the design of new multitarget drugs with great potential for the efficacious treatment of neurodegenerative disorders in which neuroinflammation and tau deposition play a prominent pathogenic role. Overall, we hope this study will facilitate medicinal chemists working in the field to spot new opportunities in multitarget anti-AD drug design, which should enhance the likelihood of discovering new drugs that are able to halt or slow down disease progression and/or more efficiently relieve the symptoms.

## Figures and Tables

**Table 1:** List of biological targets pursued in multitarget anti-AD agents and their abbreviations.

Abbreviation	Target	Abbreviation	Target
A1/A2	adenosine A1/A2 receptor	JNK1-3	c-Jun N-terminal kinases 1-3
AChE	acetylcholinesterase	KEAP1	Kelch-like ECH-associated protein 1
Aβ aggr	β-amyloid aggregation	LOX1/5/15	Lipoxygenase 1/5/15
α2	α2 adrenergic receptor	LTP	long-term potentiation
α7-nAChR	α7 nicotinic receptor	M1	muscarinic M1 receptor
α-syn	α-synuclein aggregation	MAO-A/MAO-B	monoamine oxidase A/B
BACE-1	β-secretase	miR-15b	microRNA 15b
BChE	butyrylcholinesterase	MMP2	matrix metalloproteinase-2
Biometal	biometals chelation	m-nAChR	muscle-type nicotinic receptor
CA1/CA2	carbonic anhydrase 1/2	MPO	myeloperoxidase
Casp1/Casp3	caspase 1/3	MT	microtubule
CaV	voltage-gated calcium channel	NMDA	N-methyl-D-aspartate receptor
CB1/CB2	cannabinoid receptor 1/2	NO	nitric oxide release
CDK1/CDK5	cyclin-dependent kinase 1/5	NOS	nitric oxide synthase
CK1	casein kinase 1	NRF2	nuclear factor-erythroid 2 p45-related factor 2
CLK1/3	cdc2-like kinase 1/3	OS	oxidative stress
COX1/COX2	cyclooxygenase 1/2	P2X7	purinergic P2X7 receptors
D2	dopamine D2 receptor	PARP-1	poly(ADP-ribose) polymerase 1
DYRK1/2	dual-specificity tyrosine phosphory-lation-regulated kinase 1/2	PDE4/5/9/10	phosphodiesterases 4/5/9/10
ERK	extracellular signal-regulated kinase	PHOS	serine/threonine phosphatases
ERα/ERβ	estrogen receptor α/β	PIM1	Pim1 kinase
FAAH	fatty acid amide hydrolase	PKC	protein kinase C
FYN	Fyn kinase	POP	prolyl oligopeptidase
GSK-3α/3β	glycogen synthase kinase 3α/3β	QR	quinone reductase
H1/H3	histamine H1/H3 receptor	ROCK1/2	Rho-associated coiled-coil kinase 1/2
5-HT1/2/4/6/7	serotonin receptor 1/2/4/6/7	S1	sigma-1 receptor
HDAC1-7,9	histone deacetylase 1-7,9	SERT	serotonin reuptake transporter
HIF1	hypoxia-inducible factor 1	SSAO/VAP-1	semicarbazide-sensitive amine oxidase/vascular adhesion protein-1
HSP27	heat shock protein 27	TAU	tau aggregation
IDO1	indoleamine 2,3-dioxygenase 1		

## Data Availability

Data are contained within the article and Supplementary Materials.

## Supplementary Materials

The following supporting information can be downloaded at: https://www.mdpi.com/article/10.3390/ph15050545/s1, Figure S1: Binary Combinations Map (Gephi file); Table S1: Target ID Numbers; Table S2: Binary Target Combinations.
